# Supplementary material for: A Novel Bispecific T-Cell Engager (CD1a x CD3ε) BTCE Is Effective against Cortical-Derived T Cell Acute Lymphoblastic Leukemia (T-ALL) Cells
Source: Cancers (Basel). 2022 Jun 11;14(12):2886. doi: 10.3390/cancers14122886 (PMC9221015; doi:10.3390/cancers14122886)
Supplement: Supplementary file 1 [file cancers-14-02886-s001.zip › cancers-1739895_Supplementary Materials.pdf]

cancers

MDPI

Supplementary

A Novel Bispecific T-Cell Engager (CD1a x CD3ε) BTCE Is Effective against Cortical-Derived T Cell Acute Lymphoblastic Leukemia (T-ALL) Cells

Caterina Riillo [1,†], Daniele Caracciolo [1,†], Katia Grillone [1], Nicoletta Polerà [1], Franca Maria Tuccillo [2], Patrizia Bonelli [2], Giada Juli [1], Serena Ascrizzi [1], Francesca Scionti [3], Mariamena Arbitrio [4], Mariangela Lopreiato [1], Maria Anna Siciliano [1], Simona Sestito [5], Gabriella Talarico [6], Eulalia Galea [7], Maria Concetta Galati [7], Licia Pensabene [5], Giovanni Loprete [8], Marco Rossi [1], Andrea Ballerini [9], Massimo Gentile [10], Domenico Britti [8], Maria Teresa Di Martino [1], Pierosandro Tagliaferri [1] and Pierfrancesco Tassone [1,11,*]

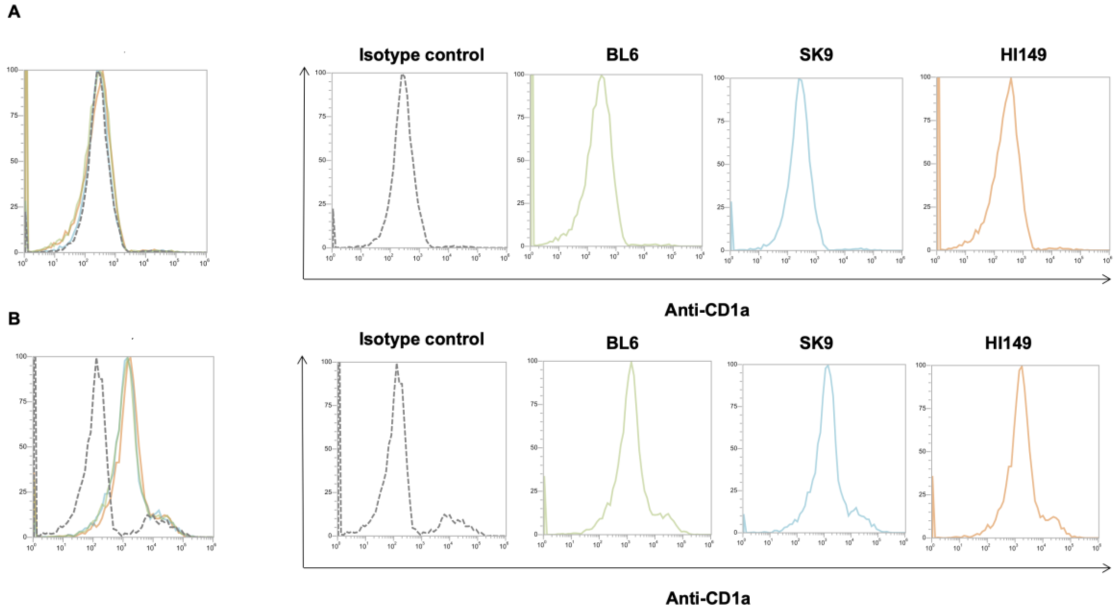

Figure S1. Anti-CD1a fluorochrome-conjugated antibodies (Bl6, SK9, HI149) staining on HEK293T EV. A) Shows representative FACS histogram of anti-CD1a fluorochrome-conjugated antibodies (Bl6, SK9, HI149) staining on HEK293T EV. B) Shows representative FACS histogram of anti-CD1a fluorochrome-conjugated antibodies (Bl6, SK9, HI149) staining on HEK293T/CD1a.

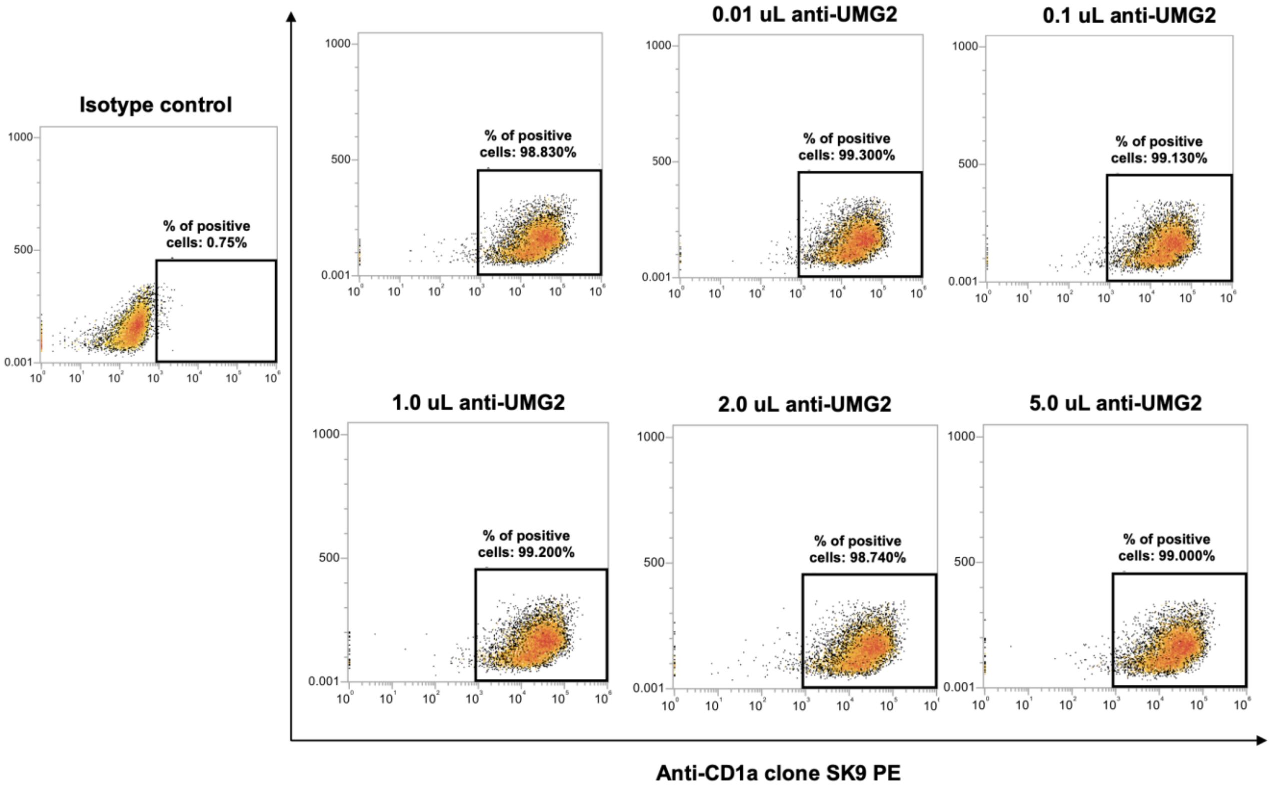

Figure S2. Competitive binding assay between anti-CD1a PE fluorochrome-conjugated antibody clone SK9 and anti-UMG2. Figure S2 shows FACS histogram of competitive binding assay between anti-CD1a PE fluorochrome-conjugated antibody clone SK9 and anti-UMG2 (unconjugated) on HPB-ALL cell line.

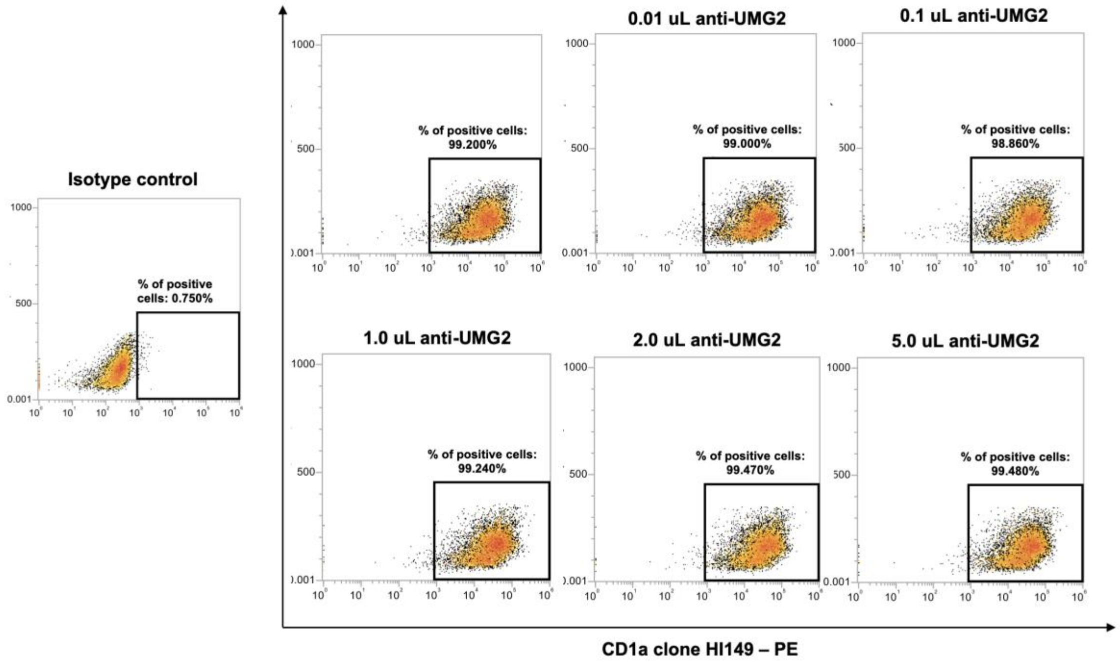

Figure S3. Competitive binding assay between anti-CD1a PE fluorochrome-conjugated antibody clone HI149 and anti-UMG2. Figure S3 shows FACS histogram of competitive binding assay

between anti-CD1a PE fluorochrome-conjugated antibody clone HI149 and anti-UMG2 (unconjugated) on HPB-ALL cell line.

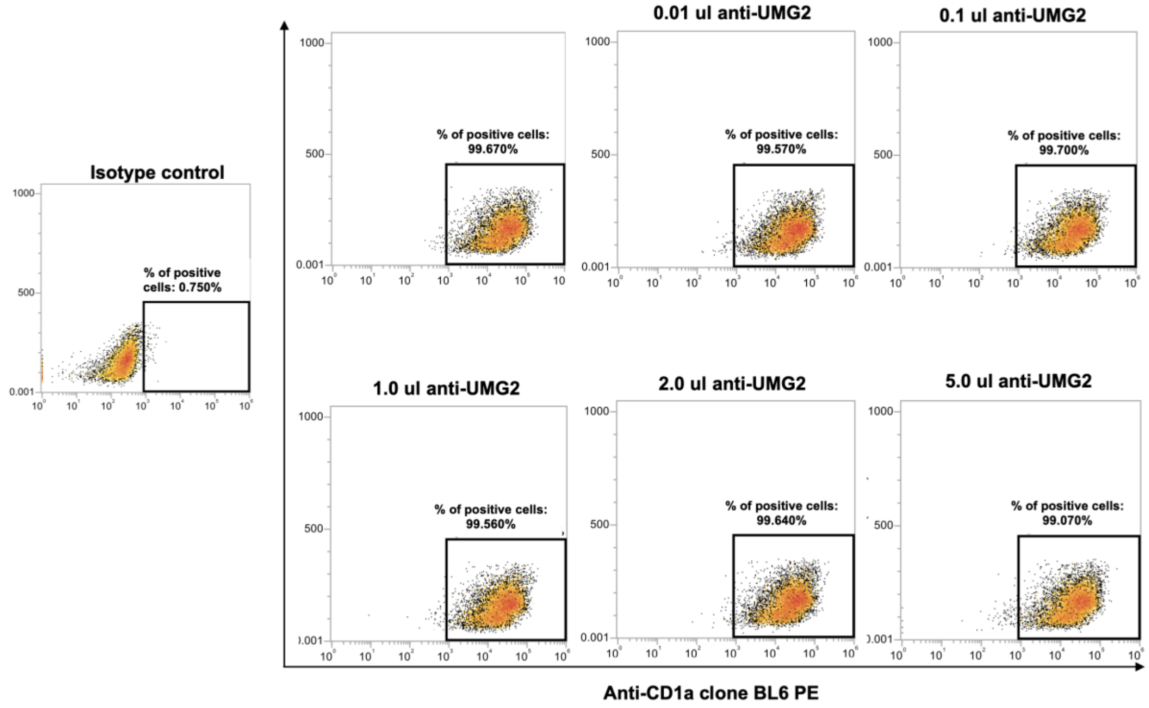

Figure S4. Competitive binding assay between anti-CD1a PE fluorochrome-conjugated antibody clone BL6 and anti-UMG2. Figure S4 shows FACS histogram of competitive binding assay between anti-CD1a PE fluorochrome-conjugated antibody clone BL6 and anti-UMG2 (unconjugated) on HPB-ALL cell line.

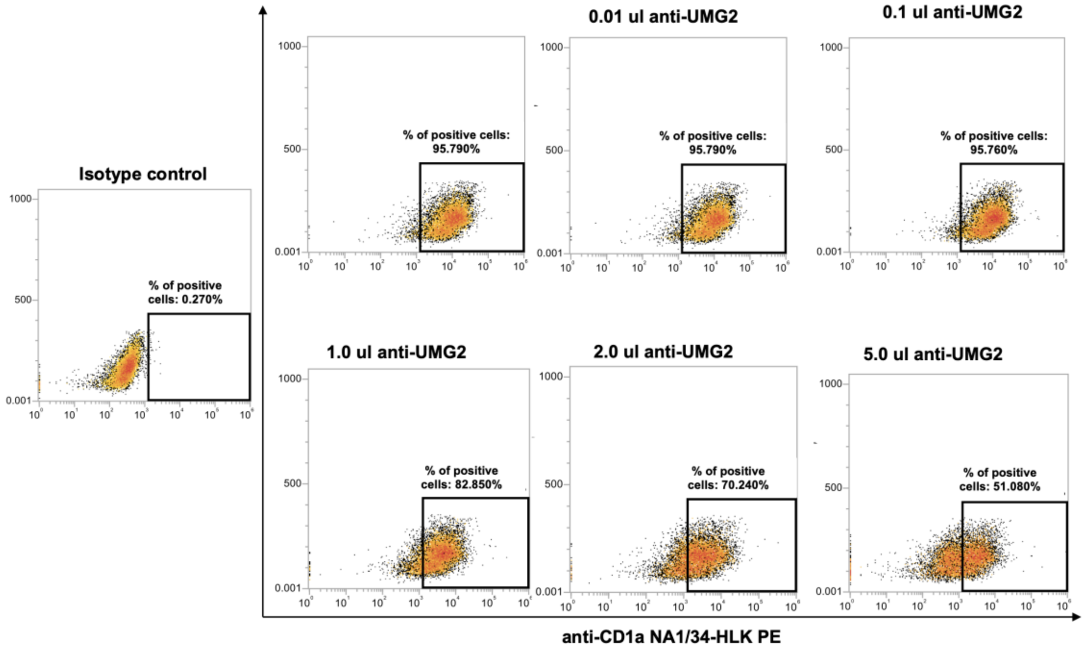

Figure S5. Competitive binding assay between anti-CD1a PE fluorochrome conjugated antibody clone NA1/34-HLK and anti-UMG2. Figure S5 shows FACS histogram of competitive binding assay between anti-CD1a PE fluorochrome-conjugated antibody clone NA1/34-HLK and anti-UMG2 (un-conjugated) on HPB-ALL cell line.

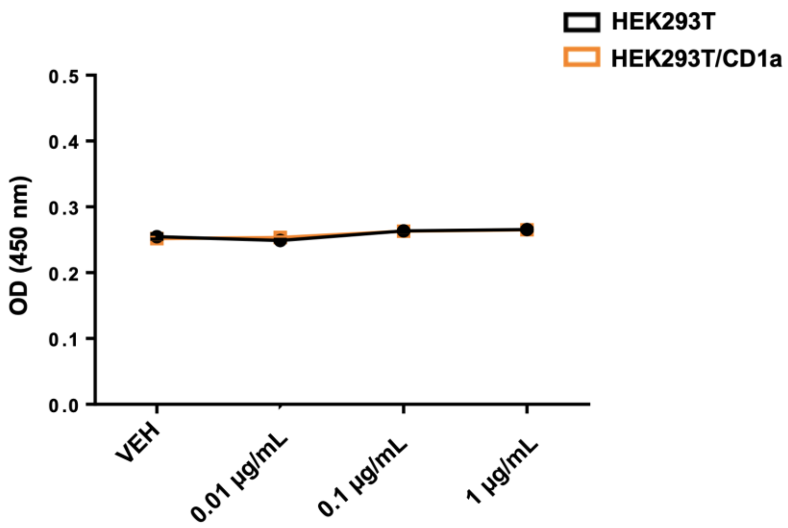

Figure S6. CD1a x CD3ε BTCE direct cytotoxicity. CD1a x CD3ε BTCE direct cytotoxicity in the absence of effector cells on HEK293T and HEK293T CD1a treated with vehicle or 0.01 µg/mL, 0.1 µg/mL and 1 µg/mL of CD1a x CD3ε BTCE.

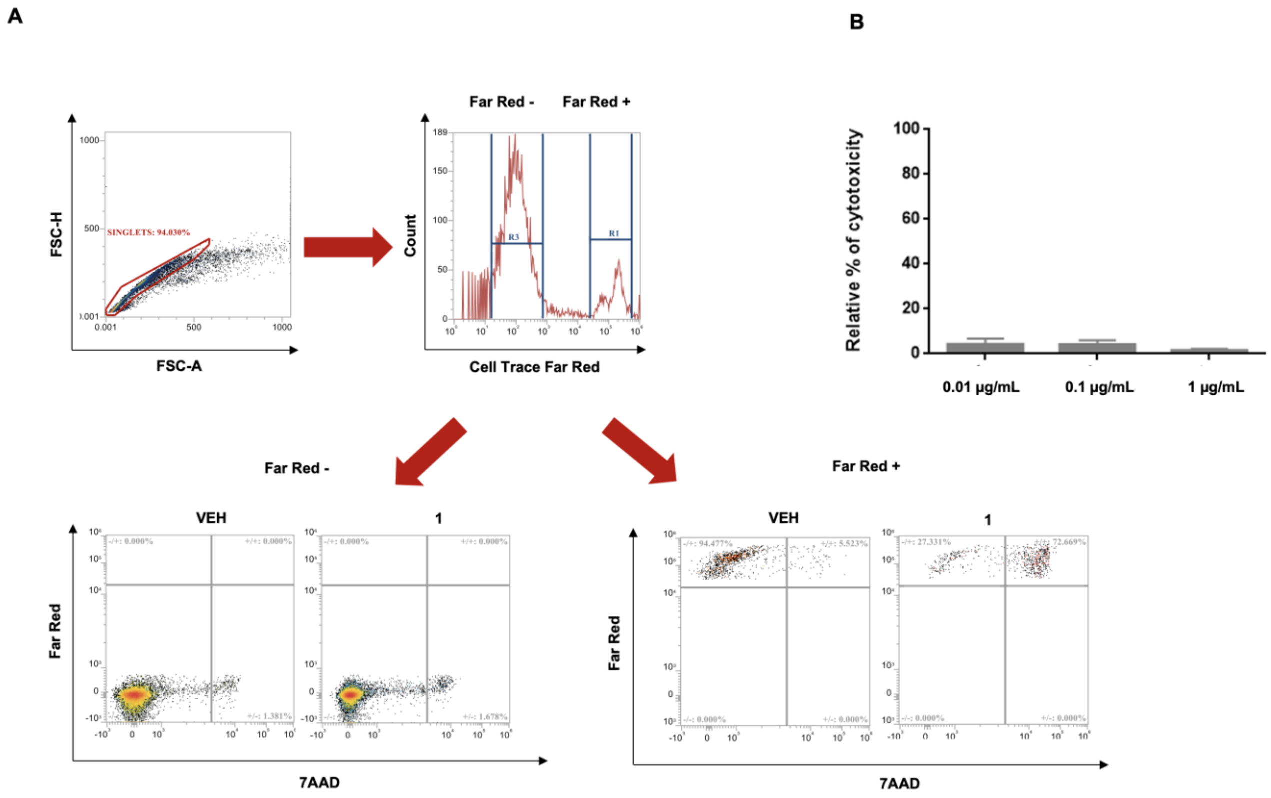

Figure S7. Flow cytometry gating strategy to evaluate cytotoxicity and CD1a x CD3ε cytotoxicity on PBMCs. Shows flow cytometry gating strategy to evaluate cytotoxicity. In detail,7-AAD-/ Far

Red+ cells were considered alive T-ALL cells while 7-AAD+/ Far Red+ were considered dead T-ALL cell. 7-AAD-/ Far Red- were considered alive PBMC while 7-AAD+/ Far Red- were considered dead PBMC.B) Relative % of cytotoxicity on PBMC effectors cells co-culture with T-ALL cells at 10:1 E:T ratio and treated with vehicle or 0.01µg, 0.1µg and 1µg of CD1a x CD3ε BTCE.

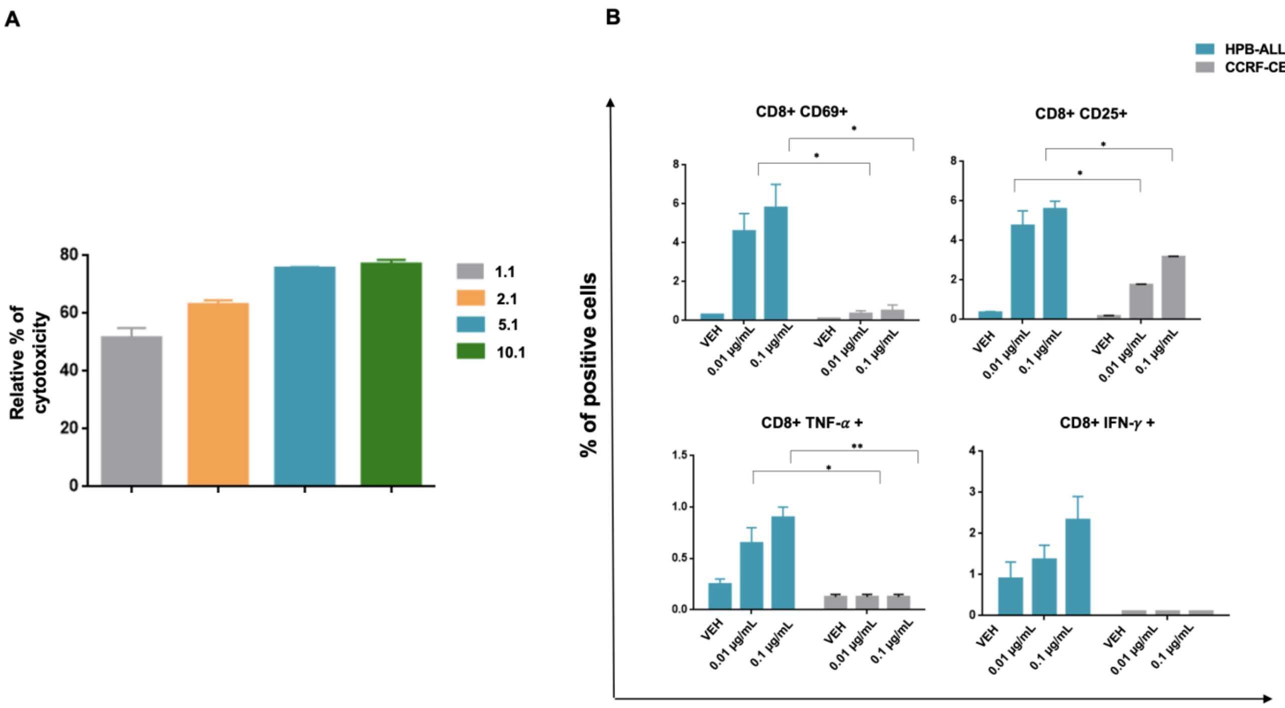

Figure S8. CD1a x CD3ε cytotoxicity at low E:T ratio and CD1a x CD3ε mediated T-cell activation against CD1a—cell line. A) Relative % of cytotoxicity against T-ALL cell line (HPB-ALL) co-cultured with PBMCs in the presence of increasing concentration (0.01 µg/mL, 0.1 µg/mL and 1 µg/mL) of CD1a x CD3 BTCE (at different effector to target (E: T). ratio (from 1:1 to 10:1) B) Expression of activation markers (CD69, CD25) and cytokine release (IFN-y, TNF-a) on T lymphocytes co-cultured with CD1a+ T-ALL cells (HPB-ALL) and CD1a- T-ALL cells (CCRF-CEM) at 10:1 E:T ratio in the presence of vehicle or increasing concentration of CD1a x CD3ε. * $p < 0.05$, ** $p < 0.01$.
